# APP intracellular domain acts as a transcriptional regulator of miR-663 suppressing neuronal differentiation

**DOI:** 10.1038/cddis.2015.10

**Published:** 2015-02-19

**Authors:** R Shu, W Wong, Q H Ma, Z Z Yang, H Zhu, F J Liu, P Wang, J Ma, S Yan, J M Polo, C C A Bernard, L W Stanton, G S Dawe, Z C Xiao

**Affiliations:** 1 Shunxi-Monash Immune Regeneration and Neuroscience Laboratories, Department of Anatomy and Developmental Biology, Monash University, Clayton, Victoria 3800, Australia; 2 The Key Laboratory of Stem Cell and Regenerative Medicine, Institute of Molecular and Clinical Medicine, Kunming Medical College, Kunming 650031, China; 3 Australian Regenerative Medicine Institute, Monash University, Clayton, Victoria 3800, Australia; 4 Department of Pharmacology, Yong Loo Lin School of Medicine, National University Health System, National University of Singapore, Singapore 119077, Singapore; 5 Neurobiology and Ageing Programme, Life Sciences Institute, National University of Singapore, Singapore 119077, Singapore; 6 Singapore Institute for Neurotechnology (SINAPSE), Singapore 117456, Singapore; 7 Institute of Neuroscience, Soochow University, Suzhou 215123, China; 8 College of Animal Science and Technology, Henan University of Science and Technology, Luoyang 471000, China; 9 Department of Pediatrics, Duke University School of Medicine, Durham, NC 27710, USA; 10 Stem Cell and Developmental Biology Group, Genome Institute of Singapore, Singapore 138672, Singapore

## Abstract

Amyloid precursor protein (APP) is best known for its involvement in the pathogenesis of Alzheimer's disease. We have previously demonstrated that APP intracellular domain (AICD) regulates neurogenesis; however, the mechanisms underlying AICD-mediated regulation of neuronal differentiation are not yet fully characterized. Using genome-wide chromatin immunoprecipitation approaches, we found that AICD is specifically recruited to the regulatory regions of several microRNA genes, and acts as a transcriptional regulator for miR-663, miR-3648 and miR-3687 in human neural stem cells. Functional assays show that AICD negatively modulates neuronal differentiation through miR-663, a primate-specific microRNA. Microarray data further demonstrate that miR-663 suppresses the expression of multiple genes implicated in neurogenesis, including *FBXL18* and *CDK6*. Our results indicate that AICD has a novel role in suppression of neuronal differentiation via transcriptional regulation of miR-663 in human neural stem cells.

Amyloid precursor protein (APP), a ubiquitous type I transmembrane receptor, is processed by *α*-, *β*- and *γ*-secretase enzymes in two distinct cascades. Processing of APP by *β*- and *γ*-secretase in the amyloidogenic cascade leads to release of amyloid beta peptide (A*β*), while in the nonamyloidogenic pathway, mediated by *α*- and *γ*-secretase, A*β* is not produced. The biological role of A*β* has been the focus of considerable research interest as it is associated with the formation of amyloid plaques in the pathology of Alzheimer's disease (AD).^1^ However, the function of the APP intracellular domain (AICD), another APP-derived cleavage product, remains incompletely understood.^2^ Over the past decades, AICD has sparked research interest for its roles in apoptosis, synaptic plasticity and neural development.^3, 4^ In addition, AICD transgenic or knock-in mutant animal models have been reported to display AD-like pathological features, such as neuronal loss, tau aggregation, neuroinflammation, impaired neurogenesis and cognitive performance.^5, 6, 7, 8, 9, 10^

MicroRNAs (miRNAs) are widely distributed, small, non-coding RNA molecules that have emerged as post-transcriptional regulators of genes involved in developmental processes and disease.^11^ In the nervous system, some miRNAs act as key post-transcriptional regulators in neurogenesis, axonal pathfinding, apoptosis and synaptic plasticity.^12, 13^ Moreover, several miRNA-profiling studies have shown that miRNA expression patterns are altered in AD brains and peripheral tissues. However, whether the changes of miRNA pattern are the cause or the consequence of the disease remains elusive.^14^

We have previously shown that transient axonal glycoprotein-1 interacts with APP as a novel ligand, and this interaction results in the inhibition of neurogenesis through an AICD-mediated action.^15, 16^ Upon processing of APP, AICD is released and translocates into the nucleus. Once AICD is in the nucleus, it can influence gene transcription.^17, 18^ A recent *in vivo* study showed that APP could regulate neurogenesis by antagonizing miR-574-5p in the developing cerebral cortex of mice.^19^ However, the molecular mechanism by which APP inhibits neural stem cell (NSC) differentiation remains to be determined.

In this study, we hypothesized that APP might influence physiological processes, such as neurogenesis, via direct binding of AICD to the miRNA-embedding genomic region. To test this hypothesis, we applied a genome-wide search for AICD-regulated miRNAs, using chromatin immunoprecipitation coupled with deep DNA sequencing (ChIP-seq), and then selected dozens of candidate miRNAs to validate their regulation by AICD as well as their role in the neuronal differentiation of human neural stem cells (hNSCs). Our findings demonstrate that AICD binds to regulatory regions of specific miRNAs in human genome and suppresses neuronal differentiation through transcriptional regulation of miR-663.

## Results

### Distribution of the AICD ChIP-seq miRNA-binding peaks

**Figure 1:**
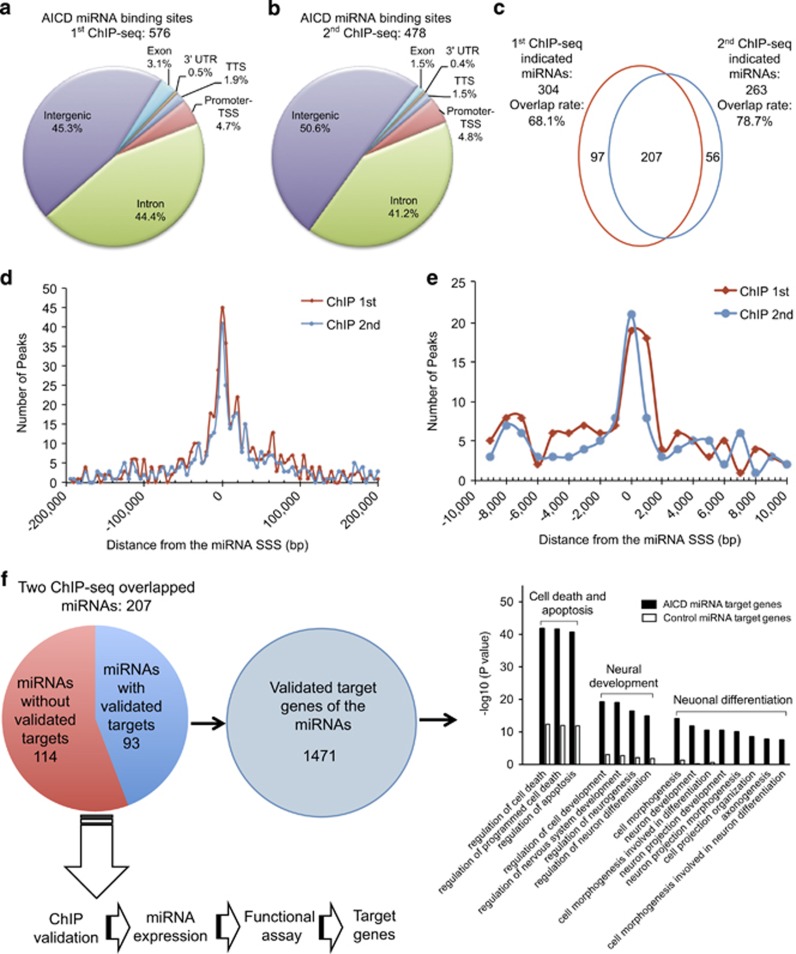
AICD is recruited to the miRNA-embedding regions in SH-SY5Y cells. (**a** and **b**) Distribution of AICD miRNA-binding peaks from the first (**a**) and second (**b**) ChIP-seq data sets in exons, introns, 3'-UTR, transcription terminal site (TTS), promoter-transcription start site (TSS) and intergenic regions of the genome. (**c**) Overlap of the miRNAs indicated by AICD-binding peaks from the first and second ChIP-seq data sets. (**d** and **e**) The distribution of the peaks within 200 kb (**d**) and 10 kb (**e**) of miRNA SSS. The *x* axis shows the base distance from the miRNA SSS, noted as 0. The *y* axis is the number of peaks counted in the regions. (**f**) Functional annotation clustering analysis of the AICD ChIP miRNAs and schematic diagram of the strategy of this study

In the genome, miRNAs are located either between independent transcription units (intergenic), or in the intronic or exonic regions of genes. The intergenic miRNAs are transcribed independently, whereas the intronic and exonic miRNAs may be transcribed with their host genes. To comprehensively identify AICD-binding sites within the promoter regions of miRNAs, duplicate ChIP-seq experiments were performed in SH-SY5Y cells. The AICD-binding sites generated from the two data sets were mapped on the genome relative to the nearest miRNAs and annotated with respect to their distance from the miRNA stem–loop start sites (SSS; Supplementary Tables S1 and S2). Analysis of the pooled data showed that AICD binds to 576 sites corresponding to 304 miRNAs in set 1, and 478 sites corresponding to 263 miRNAs in set 2, with an overlap of 207 miRNAs (Figures 1a–c). These results suggest that the binding between AICD and miRNA regions is highly reproducible through the ChIP-seq assays. Notably, most reported AICD-regulated genes were also found in our ChIP-seq data, representing robust controls for verifying the reliability of our ChIP-seq data (Supplementary Table S3).

Genome analysis of the ChIP-seq data shows that most AICD-binding sites for miRNAs are enriched in intergenic and intronic regions, but not in the promoter regions of protein-coding genes (Figures 1a and b), as most of the miRNAs are located in the intergenic or intronic regions.^20^ As we know, nearly all the intergenic miRNAs and ~35% of the intronic miRNAs, which consist of the majority of miRNAs, have their own promoters,^21, 22^ whereas the ChIP-seq-binding sites in our study are not randomly distributed but are enriched in the proximal miRNA SSS (Figures 1d and e). Taken together, our data indicate that AICD binds to the upstream regulatory elements of these miRNAs.

### Functional annotation of the AICD-regulated miRNAs

Within the 207 miRNAs identified in the two ChIP-seq data sets, 93 miRNAs are associated with 1471 validated target genes according to the miRWalk database (Supplementary Table S4).^23^ Meanwhile, 93 randomly selected miRNAs, which were not in our ChIP-seq data sets, were used as controls. Using functional annotation clustering (FAC), we compared the enrichment of gene ontology terms between the AICD and control miRNA group.^24^ The outcome suggests that the best-known functions of AICD are enriched in the AICD-regulated miRNAs (Figure 1f and Supplementary Table S5). Therefore, we hypothesized that AICD may perform its roles through regulating miRNAs. To test this hypothesis, these miRNAs should be validated step-by-step through ChIP, miRNA expression and functional assays, as well as target gene identification.

### Validation of selected AICD-regulated miRNAs using ChIP

**Figure 2:**
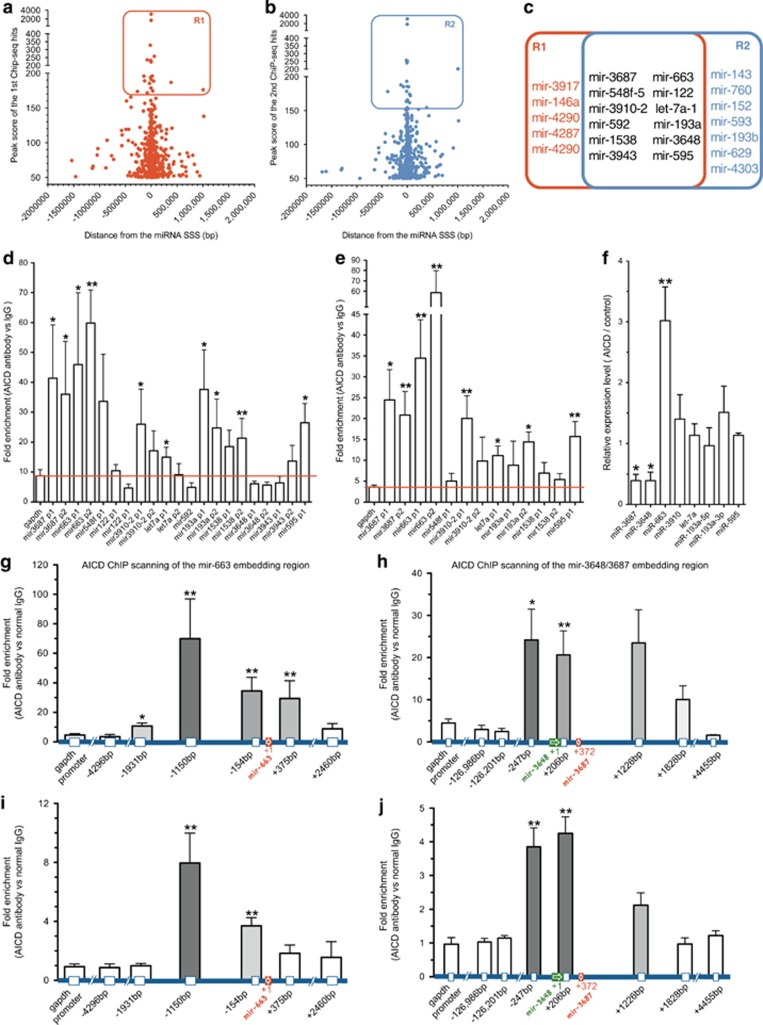
ChIP validation and miRNA expression assay for possible miRNAs regulated by AICD in hNSCs. (**a** and **b**) Top 20 'peak score' miRNA hits were selected from the first (R1; **a**) and the second ChIP-seq data set (R2; **b**). (**c**) The overlapping 12 miRNA hits from R1 and R2 were selected for ChIP validation. (**d**) AICD ChIP validation performed in SH-SY5Y cells overexpressing AICD. The *gapdh* promoter region is set as control. (**e**) AICD ChIP validation performed in hNSCs overexpressing AICD. (**f**) Expression fold change of the eight miRNAs in hNSCs after AICD overexpression. (**g**) AICD ChIP scanning of the mir-663-embedding region in hNSCs overexpressing AICD. The *gapdh* promoter and other regions are set as control. The *x* axis shows the base distance from the miRNA stem–loop start site, noted as +1. (**h**) AICD ChIP scanning of the mir-3648- and mir-3687-embedding regions in hNSCs overexpressing AICD. (**i** and **j**) ChIP validation of the endogenous interaction of AICD with mir-663 (**i**), mir-3648 and mir-3687 (**j**) embedding regions in wild-type hNSCs. Quantification data were analyzed from at least three independent experiments (mean±S.E.M). *P<0.05; **P<0.01

To select high-priority candidates, the miRNAs from the two ChIP-seq data sets were ranked by 'peak score' (Figures 2a and b; Supplementary Tables S1and S2). From the top 20 binding sites of the two sets (R1: Figure 2a; R2: Figure 2b), 12 miRNAs were found in common and were selected for further evaluation (Figure 2c and Table 1). Given that the regulation of gene expression through AICD is affected by the chromatin status of cell types,^25^ we chose the ReNcell VM (RVM) hNSC,^26^ a more physiological relevant cell type, for further study of the neurogenesis. To confirm AICD-specific binding at the genomic locations of the 12 miRNA genes (Table 1), we performed independent ChIP-PCR experiments in SH-SY5Y cells and hNSCs using another high specificity anti-AICD antibody (Supplementary Figure S1). These ChIP-PCR assays, using primer pairs for the 12 specified gene locations, confirmed that seven chromatin regions relevant to mir-3687, mir-3648, let-7a-1, mir-663, mir-3910, mir-193a and mir-595, were significantly enriched in both cell types (Figures 2d and e). Thus, these data demonstrate that AICD interacts with the chromatin regions of mir-3648, mir-3687, let-7a-1, mir-663, mir-3910, mir-193a and mir-595.

### AICD modulates the expression of miRNAs in hNSCs in a dual regulatory manner

AICD has been previously shown to contribute to the regulation of gene expression in a dual regulatory manner, both increasing and decreasing the expression of various genes.^3^ To reveal the biological significance of AICD-regulated miRNAs, we transduced AICD expression vector into hNSCs using a lentiviral vector and assessed the expression of the miRNAs using quantitative PCR (qPCR). After overexpression of AICD, the expression levels of miR-663 significantly increased, and those of both miR-3648 and miR-3687 were significantly decreased (Figure 2f). These results demonstrate that AICD may modulate the expression miRNAs in a dual regulatory manner.

As the binding sites for mir-663, mir-3648 and mir-3687 were situated near to the SSS of these miRNAs, the embedded regions were scanned using ChIP in both AICD-overexpressed and wild-type hNSCs. We found that the index of AICD-binding enrichment was significantly increased at regions of −1150, −154 and +375 bp away from the mir-663 SSS, but not at more distant regions or the control *gapdh* promoter region (Figure 2g). The mir-3648 and mir-3687 were clustered together in the genome, and the index of enrichment was significantly increased at −247 and +206 bp from the mir-3648 SSS, but not at more distant regions or *gapdh* promoter region (Figure 2h). Similarly, ChIP-PCR validations were performed in wild-type hNSCs, and the result confirmed the interactions between the endogenous AICD and these regions (Figures 2i and j). Because the *cis*-acting elements are indicators of promoter activity, we questioned whether these two AICD-binding regions could be regulatory regions for these miRNAs. To this end, the distribution of Encyclopedia of DNA Elements (ENCODE) sites and CpG islands were retrieved from the UCSC Genome Browser.^27^ We found that the ENCODE sites and CpG islands were enriched in these regions. In addition, the distributions of ENCODE peaks were highly consistent with the distribution of our AICD ChIP enrichments in these regions (Supplementary Figure S2). Therefore, these data indicate that AICD is recruited to the transcriptional regulatory regions of these miRNA genes.

### AICD suppresses the neuronal differentiation of hNSCs

**Figure 3:**
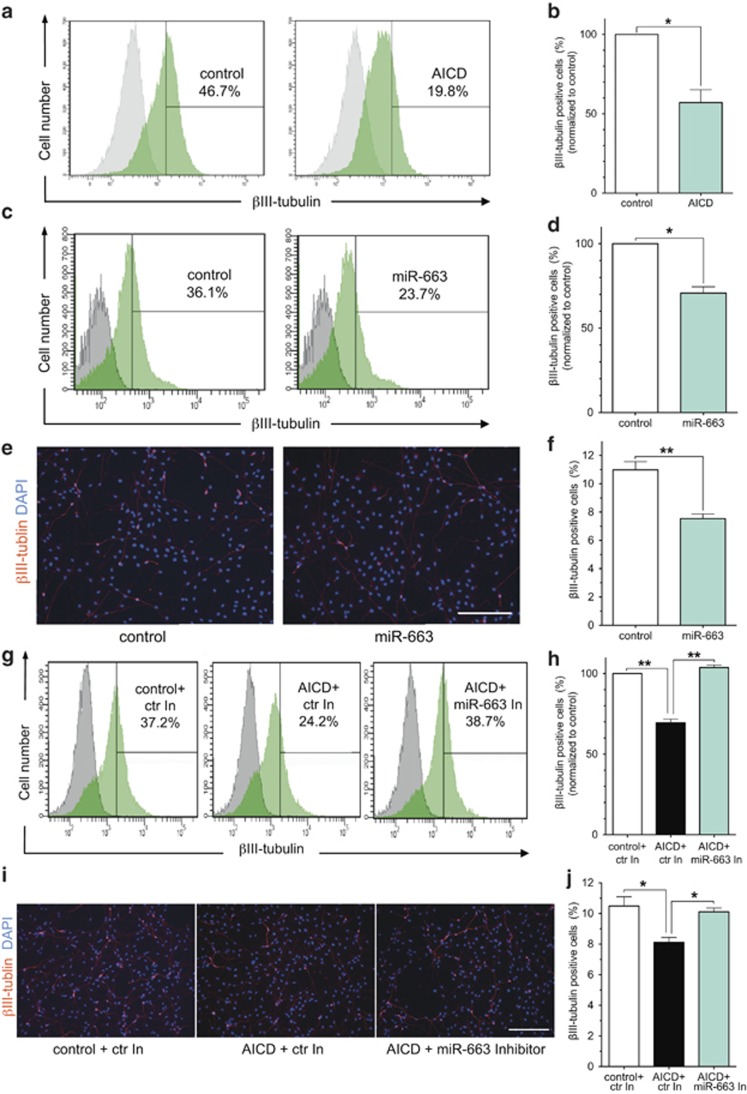
AICD inhibits hNSC differentiation through miR-663. (**a** and **b**) Representative histograms (**a**) and quantification (**b**) of *β*III-tubulin detection using flow cytometry in differentiated hNSCs transfected with AICD and control (green: *β*III-tubulin; gray: isotype control). (**c** and **d**) Representative histograms (**c**) and quantification (**d**) of *β*III-tubulin detection using flow cytometry in differentiated hNSCs transfected with miR-663 mimics and control (green: *β*III-tubulin; gray: isotype control). (**e** and **f**) Representative images (**e**) and quantification (**f**) of *β*III-tubulin detection using immunohistochemistry in differentiated hNSCs transfected with miR-663 mimics and control. Scale bar=200 *μ*m. (**g** and **h**) Representative histograms (**g**) and quantification (**h**) of *β*III-tubulin detection using flow cytometry in differentiated hNSCs transfected with control vector plus non-targeting control inhibitors (ctr In), AICD plus non-targeting control inhibitors, AICD plus miR-663 antisense inhibitors, respectively (green: *β*III-tubulin; gray: isotype control). (**i** and **j**) Representative images (**i**) and quantification (**j**) of *β*III-tubulin detection by immunohistochemistry in differentiated hNSCs transfected with control vector plus non-targeting control inhibitors (ctr In), AICD plus non-targeting control inhibitors, AICD plus miR-663 antisense inhibitors, respectively. Scale bar=200 *μ*m. Quantification data were analyzed from at least three independent experiments (mean±S.E.M). *P<0.05; **P<0.01

We have previously reported that AICD negatively regulates the neurogenesis of mouse NSC,^15^ a finding that was also supported by studies in the AICD transgenic mouse model.^6^ However, data from AD patients and a number of mouse AD models have revealed apparent conflicts with respect to whether neurogenesis is up- or downregulated.^28^ To investigate whether AICD restricts human neuronal differentiation, we overexpressed AICD in hNSCs, and we found that the number of neuronal-like cells in the AICD-transfected group was significantly decreased in comparison with the control group as demonstrated by expression of the neuronal marker *β*III-tubulin (Figures 3a and b).

### miR-663 suppresses neuronal differentiation of hNSCs

To determine which of the AICD-regulated miRNAs are involved in inhibiting neuronal differentiation, we transfected molecular mimics of miR-663, miR-3687 or miR-3648 into hNSCs. After differentiation, the transfected hNSCs were assessed for neuronal differentiation by flow cytometry and immunohistochemistry, using antibodies against *β*III-tubulin. Flow cytometry analysis showed that the number of *β*III-tubulin-positive cells was significantly decreased in cells overexpressing miR-663 (Figures 3c and d), but not in those overexpressing miR-3648 or miR-3687 (Supplementary Figures S3a and b). Similar results were obtained by quantitative analyses of the immunohistochemistry (Figures 3e and f, Supplementary Figures S3c and d). We also estimated the percentage of *β*III-tubulin-positive cells after transfection of the corresponding antisense inhibitors and the percentage of GFAP (an astroglial marker)-positive cells after transfection with the miRNAs; however, no significant changes were detected (Supplementary Figures S3e–h). These results demonstrate that, among the AICD-regulated miRNAs, miR-663 mediates suppression of neuronal differentiation in hNSCs.

To further confirm that AICD inhibits neuronal differentiation through miR-663, we investigated whether inhibition of miR-663 could rescue AICD-mediated suppression of neuronal differentiation. hNSCs were co-transfected with miR-663 antisense inhibitor and AICD. After differentiation, both flow cytometry and immunohistochemistry showed that the number of *β*III-tubulin-positive cells was significantly increased after co-transfection of miR-663 antisense inhibitor with AICD (Figures 3g–j). These observations indicate that miR-663 is a mediator for the AICD suppression of neuronal differentiation in hNSCs.

### miR-663 targets multiple genes in hNSCs

**Figure 4:**
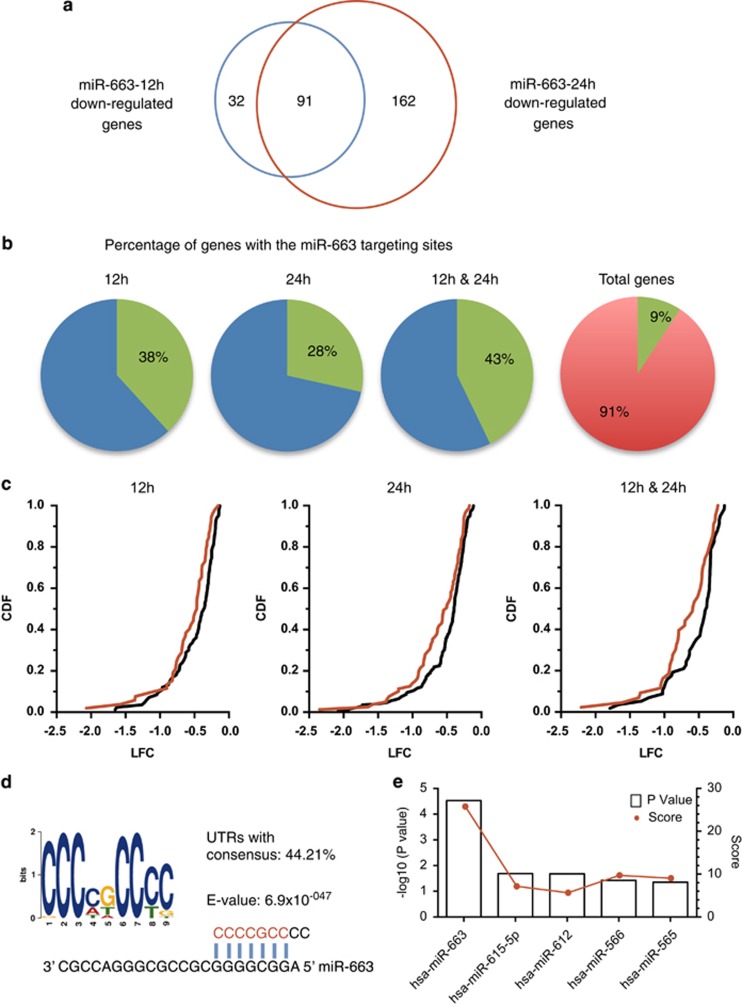
Multiple genes are downregulated after miR-663 mimics transfection in hNSCs. (**a**) Ninety-one genes are downregulated significantly downregulated (*P*<0.001) at both 12 and 24 h after miR-663 transfection. (**b**) Percentage of the genes containing miR-663 target sites predicted by Targetscan in the miR-663 microarray downregulated genes and total genes. (**c**) Cumulative distribution plots of log2-transformed gene expression fold changes (LFCs) for downregulated genes containing miR-663 target sites predicted by TargetScan (red) and all other downregulated genes (gray) after miR-663 transfection. The *y* axis shows cumulative distribution function (CDF) of LFC distribution. (*P*-values<0.05 by Wilcoxon test). (**d**) Representative motif found by MEME in the 3′-UTRs of the genes downregulated at both 12 and 24 h. The sequence logo of the weight matrix was constructed by MEME. (**e**) DIANA-mirExTra algorithm shows miR-663 hemxamers are enriched in the 3′-UTRs of the genes downregulated at both 12 and 24 h. The *P*-values and scores of the top five most significant miRNAs calculated with DIANA-microT are shown

Vertebrate miRNAs have been reported to target multiple genes at the transcriptional level through interaction with untranslated regions (UTRs) to perform their biological function.^29, 30, 31^ Interestingly, miR-663, a member of primate-specific miRNA family, has recently been found to be associated with cancer, through the regulation of different target genes in cancer cells.^32, 33^ To identify the genes targeted by miR-663 in hNSCs, the miR-663 mimics were transfected into hNSCs, and mRNA was purified and profiled on gene expression microarrays (Supplementary Figure S4a). We found that 123 and 253 genes were significantly downregulated (*P*<0.001) at 12 and 24 h after miR-633 transfection, respectively. In addition, 91 genes were significantly downregulated at both 12 and 24 h (Figure 4a and Supplementary Table S6).

In order to predict and identify the possible target genes of miR-663 among the genes with differential expression, we checked whether any are predicted targets using Targetscan.^29^ We found that the predicted direct targets of miR-663 were enriched in the downregulated genes as compared with the total genes in the study, and greatly enriched in the 91 downregulated genes (Figure 4b). Furthermore, we observed significantly over-representation of gene inhibition among the Targetscan predicted genes as compared with all the other downregulated genes (Figure 4c).^34^ To evaluate the microarray-downregulated genes solely based on the sequence motifs of their 3′-UTRs, the motif discovery tool MEME was used to computationally search the 3′-UTR sequences of the downregulated genes.^35^ We found a highly significant consensus sequence 'CCCCGCCCC' in the 3′-UTR sequences of the downregulated transcripts. This consensus motif was complementary to the positions 2–8 from the 5′ end of miR-663, the crucial seed region required for target recognition (Figure 4d).^31^ Moreover, DIANA-mirExTra hexamer analysis confirmed that the direct targets of miR-663 were enriched in the 91 downregulated genes (Figure 4e).^36^

### AICD/miR-663-downregulated genes inhibit neuronal differentiation

**Figure 5:**
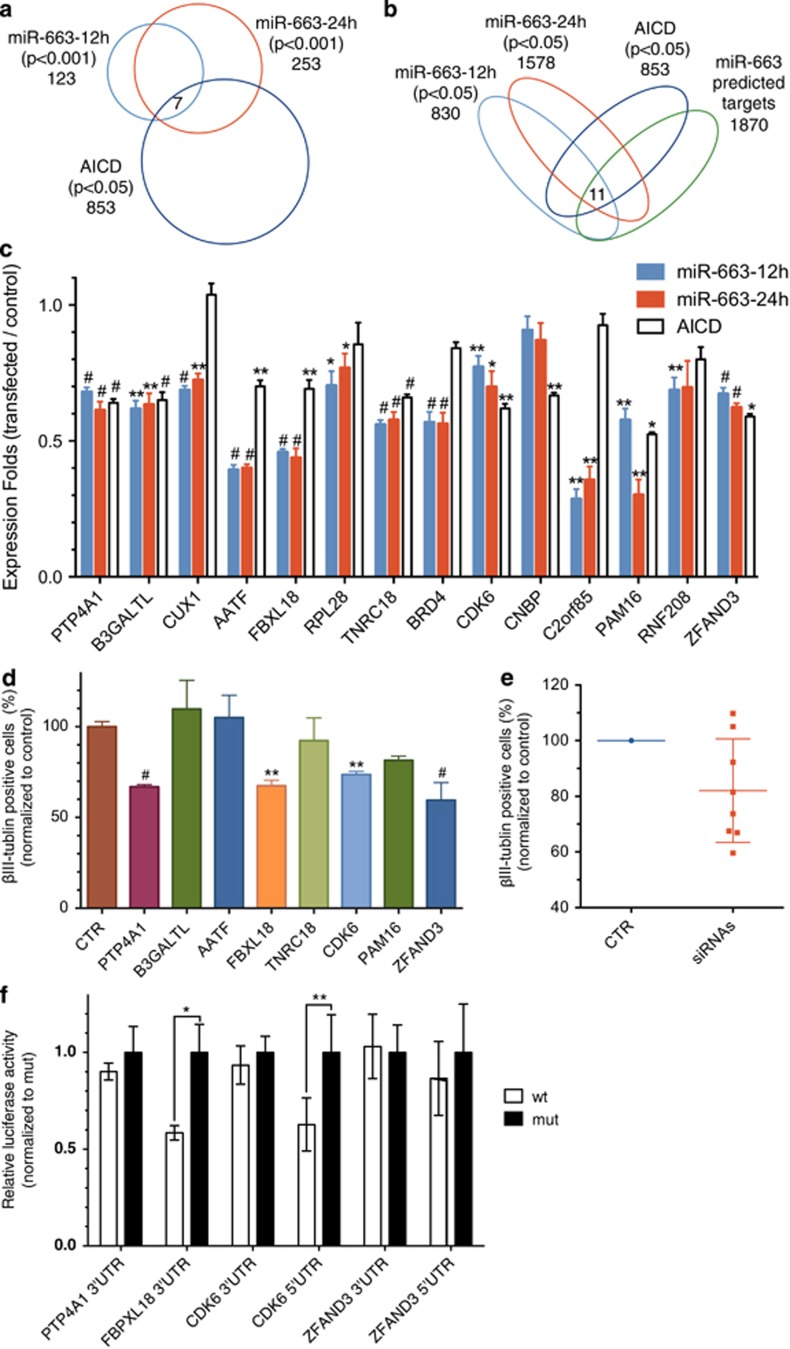
Genes downregulated by both miR-663 and AICD are associated with neurogenesis. (**a**) Three-way Venn diagram demonstrating the overlap among the miR-663 (*P*<0.001) and AICD (*P*<0.05) microarray-downregulated genes. (**b**) Four-way Venn diagram demonstrating the overlap among the miR-663 (*P*<0.05), AICD (*P*<0.05) microarray-downregulated genes and genes with miR-663 target sites predicted by Targetscan. (**c**) Validation of the gene expression fold change using qPCR. (**d**) Quantitative flow cytometry analysis of *β*III-tubulin expression in differentiated hNSCs transfected with siRNAs and non-targeting control. (**e**) Overall effects of the downregulation of these genes by siRNA on neuronal differentiation. (**f**) Dual-luciferase reporter assay shows that miR-663 directly represses the expression luciferase genes bearing 5′-UTR segments of CDK6 and 3′-UTR segments of FBPXL18. Quantification data were analyzed from at least three independent experiments (mean±S.E.M). *P<0.05, **P<0.01, ^#^*P*<0.001

To identify downstream genes of AICD/miR-663 signaling during neurogenesis, we performed gene expression microarray analysis in AICD-overexpressing hNSC cells. In brief, hNSCs were transduced by AICD, and EGF and bFGF were withdrawn to stimulate cell differentiation at 48 h post transduction, when miR-663 was upregulated (Figure 2f). After another 24 h, total RNA was prepared for microarray analysis. We found that 853 genes were significantly downregulated (*P*<0.05) after AICD overexpression (Supplementary Figure S4 and Table S7). Subsequently, we intersected the miR-663-downregulated gene profiles (*P*<0.001) with the AICD-downregulated gene expression profile, and revealed that seven genes were downregulated by both AICD and miR-663 (Figure 5a). In addition, we overlapped the miR-663-downregulated gene profiles (*P*<0.05; Supplementary Figure S4 and Table S8) with both the AICD-downregulated gene expression profile and the Targetscan-predicted genes of miR-663, and revealed that another 11 genes were downregulated by both AICD and miR-663 (Figure 5b). In total, we found 13 genes for further validation (Figure 5c). After qPCR validation, eight genes were selected for further flow cytometry analysis of neuronal differentiation, and we found that siRNA knockdown of *PTP4A1*, *FBXL18*, *CDK6* and *ZFAND3* resulted in significant suppression of neuronal differentiation (Figure 5d). In addition, the overall effect of knockdown of these genes was the inhibition of neurogenesis in the hNSCs (Figure 5e). To further determine which genes are the direct targets of miR-633, wild-type and mutant UTR segments possessing the seed matches from these four genes were cloned into dual-luciferase vectors. When co-transfected with the miR-663, repression of the reporter gene was observed in the 3′-UTR of the *FBXL18* and 5'-UTR of the *CDK6* in comparison with the corresponding mutant UTRs (Figure 5f). The reporter assay result demonstrates that AICD/miR-663 can downregulate the expression of *FBXL18* and *CDK6* directly.

## Discussion

**Figure 6:**
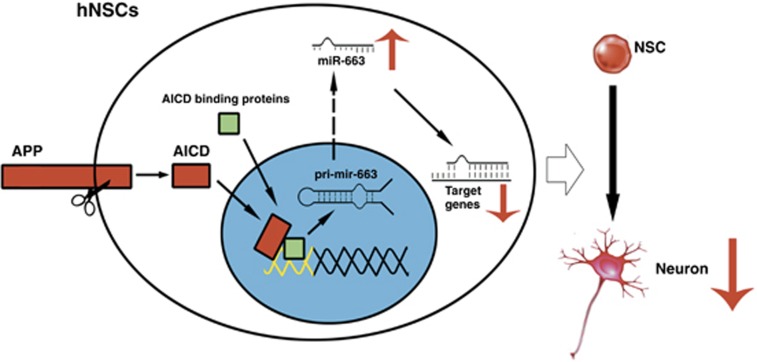
Proposed model of the APP-AICD-miR-663 signaling in hNSC differentiation. APP is located at the plasma membrane of hNSCs. Ligand binding to APP triggers an intramembrane cleavage of APP to generate the AICD, which translocates to the nucleus. Nuclear AICD promotes the expression of miRNAs, such as miR-663, which suppress the expression of genes such as *FBXL18* and *CDK6*, to inhibit hNSC neuronal differentiation

In the present study, using genome-wide ChIP-seq we have identified a set of miRNAs regulated by AICD. Furthermore, we have shown that AICD acts as a transcriptional regulator to modulate the expression of miRNAs. Importantly, we have demonstrated that miR-663 has an important role in suppressing neuronal differentiation of hNSCs *in vitro* and have identified an AICD downstream mechanism suppressing neurogenesis in hNSCs (Figure 6).

APP is considered having a key role in the pathogenesis of AD, as cleavage of APP produce A*β* peptide that is deposited in the brain of AD patients. In addition to A*β*, the ectodomain of APP has been reported to have neuroprotective effects and mediate the physiological functions of APP.^16^ The intracellular domain of APP is also believed to be functionally important, as numerous proteins interact with this region to regulate the processing and function of APP.^8, 37^ However, there is controversy over the transcriptional regulation of AICD. AICD has been first implicated with transcriptional activity through the Gal4 reporter assay.^17^ Following studies have demonstrated that AICD regulates the transcription of genes, including *LRP1*, *KAI1*, *GSK-3β*, *neprilysin* and *EGFR*.^4^ Our results, we believe, resolved some of these discrepancies by providing clues that AICD interacts with the chromatin directly to affect transcription in both of the overexpression and endogenous systems; nevertheless, the signal transduction model of AICD and its relation with the binding proteins need to be further explored. In addition, the APP family proteins, APLP1 and APLP2, also have similar C terminus as APP.^38^ A recent study has shown the nuclear signaling function for APP, and APLP2 is absent in APLP1.^39^ However, the specific interactions of these proteins with chromatin, and the differences of nuclear function among the APP family members, need to be determined in the future.

As miRNAs are important regulators of gene expression, we focus on the possibility that AICD modulates the expression of miRNAs. In search for the miRNAs regulated by AICD, we performed ChIP-seq experiments in APP695-overexpressing cells, given that AICD turns over rapidly, whereas it is preferentially produced from the APP695 isoform.^40^ It is established that miRNAs are first transcribed as primary miRNAs (pri-miRNAs).^21, 22^ As the pri-miRNAs vary in length from a few hundred bases up to hundreds of kilobases, the regulatory regions for the miRNAs could be located up to several megabases from the miRNA stem–loop sequence in the genome.^41, 42^ Consequently, a wider distance criterion ranging within 2 megabases from the SSS was used to associate the ChIP-seq peaks with miRNAs. Using our ChIP-seq data, we discovered a set of AICD-regulated miRNAs, and the subsequent FAC analysis suggested a promising potential that AICD may regulate apoptosis and neurogenesis through miRNAs. However, the wider distance criterion, together with the overexpression system, could result in nonspecific binding sites in the ChIP-seq data. For this reason, both ChIP validation of endogenous binding and functional validation are required in the specific cells before drawing any conclusions.

*In vivo* neuronal development is a well-orchestrated process containing several specific cell stages, such as immature precursor, neuronally committed precursor, immature neuron and mature neuron.^43^ A number of miRNAs have also been demonstrated to regulate neural stem cell proliferation, differentiation and maturation during the neuronal development.^44^ miR-663, expressed only in *Homo sapiens, Macaca mulatta* and *Pantroglodytes*, belongs to the primate-specific miRNAs.^45^ The function of miR-663 has been reported to be mainly associated with cancer biology. In acute myeloid leukemia patients, the miR-663 promoter is hypermethylated and influences leukemia cell differentiation.^46, 47^ In addition, miR-663 targets the expression of multiple genes, such as *TGFβ1, p21, H-ras, eEF1* and *HSPG2*, to regulate cancer cell proliferation and chemotherapy resistance.^32, 33, 48, 49, 50^ Moreover, miR-663 is related to smooth muscle cell phenotypic switch in the cardiovascular system.^51^ Briefly, these previous studies provide a clue that miR-663 is able to influence cell proliferation and differentiation; however, its role in the nervous system is not well understood. Our findings show that miR-663 can decrease the number of *β*III-tubulin-positive cells during neuronal differentiation. Given that miR-663 might influence the proliferation of hNSCs, we performed a BrdU incorporation assay as well; however, no significant differences were found (Supplementary Figure S5a and b). We also evaluated the morphology of differentiated neurons after miR-663 overexpression by measuring the length of neurites. Preliminary result showed that miR-663 did not decreased the length significantly (Supplementary Figure S5c). Therefore, the exact role of miR-663 at different stages of the neuronal differentiation needs to be explored in future studies.

As multiple different miR-663 target genes have been reported, we hypothesized that miR-663 also targets multiple genes to perform its function in the nervous system. Using gene expression microarrays, we found that multiple genes with miR-663 predicted that target sites were downregulated in hNSCs. After validation, we identified two direct target genes, *FBXL18* and *CDK6*, which could contribute to the AICD/miR663 inhibition of neurogenesis. *FBXL18* is a member of F-box protein family, which could form the SKP1-CUL1-F-box protein (SCF) complex to act as a protein ubiquitin ligase,^52^ and some of the SCF complexes have already been suggested to control neuronal differentiation,^53, 54^ yet the exact role of *FBXL18* remains to be demonstrated. The other gene, *CDK6*, is essential for the expansion of neuronally committed precursors and the production of newborn neurons.^43^ In addition, *CDK6* is a mediator of PAX6's modulation of cortical progenitor cell proliferation.^55^ Therefore, it is possible that miR-663 inhibits neuronal differentiation through *CDK6*.

The current study demonstrates that the abnormal processing of APP might lead to aberrant AICD regulation of miRNA expression, which may be linked to abnormal intracellular signaling. However, to address the relevance of miRNAs with AD, further studies performed *in vivo* or in human tissue are required. It is also important to acknowledge that only a small fraction of miRNA candidates were selected according to the peak-calling score and distance in this study. Interestingly, the recently identified miR-574-5p, which is associated with the neuronal inhibition by APP,^19^ is also included in both of the two data sets of our ChIP-seq miRNAs (Supplementary Tables S1 and S2). However, the direct binding of AICD to the genomic region surrounding miR-574 remains to be determined. Thus, for the remaining miRNAs, their regulation by AICD and biological roles need to be further defined in specific cell types and biological assays. Considering the recent concern on the confidence of the miRNA in the miRBase, the miRNA candidates could also be filtered according to the confidence of the miRNA in the future.^56, 57^

In conclusion, the present study has identified the role of AICD-regulated miR-663 in hNSC differentiation and provided one molecular mechanistic insight into AICD signaling in human neurogenesis *in vitro*. This may have ramifications in the context of the physiological role of APP, as well as more broadly in AD research. Further understanding of these mechanisms might shed light on the cellular processes of neurodegenerative diseases and may offer an opportunity for pharmacological intervention.

## Materials and Methods

### ChIP-Seq

ChIP-seq was performed as previously described.^58^ A SH-SY5Y cell line overexpressing *APP* was generated by the stable transfection of a pcDNA4 construct carrying *APP*. Nuclear lysates were cross-linked with 1.5 mM dithiobis succinimidyl propionate (DSP) and 1% formaldehyde. Chromatin for the immunoprecipitation was sheared to fragments below 500 bp. Quality-control checks on the DNA were run using the Agilent Bioanalyzer 2100 (Mulgrave, VIC, Australia). Antibody against C-terminal APP (BR188 from M. Goedert, University of Cambridge, UK)^25^ and control rabbit IgG were used. Immunoprecipitated DNA from ChIP experiments was subjected to deep DNA sequencing. The reads were aligned against the hg19 Human Genome Assembly (GRCh37, February 2009) and peak calling was performed using the MACS (Model-based Analysis for ChIP-Seq) software.^59^ Significant binding sites within 2 megabases from the miRNA SSS were selected as potential candidates.

### Cell culture and transfection

The SH-SY5Y cell line was grown in Dulbecco's modified Eagle's medium (DMEM; Gibco, Mulgrave, VIC, Australia), supplemented with 10% heat-inactivated fetal bovine serum (Gibco), 1% penicillin/streptomycin (Gibco). The RVM hNSC line (Millipore, Bayswater, VIC, Australia) was cultured and differentiated according to the published protocols.^26^ For proliferation, RVM cells were cultured in the RVM medium (DMEM/F12 medium (Gibco) with B27 (Gibco), 10 U/ml heparin (Sigma-aldrich, St. Louis, MO, USA) and 1% Gentamicin (Gibco)) with 20 ng/ml EGF (Peprotech, Rocky Hill, NJ, USA) and 10 ng/ml bFGF (Peprotech). For differentiation, the cells were seeded on laminin (Invitrogen, Mulgrave, VIC, Australia)-coated plates, the EGF and bFGF were removed, and 1 mM dibutyrl-cAMP (Calbiochem, Lafayette, CO, USA) and 2 ng/ml GDNF (Peprotech) were added to the differentiation media. The miRNA mimics and heparin inhibitors (Dharmacon, Lafayette, CO, USA) as well as the siRNAs (Silencer Select validated and pre-designed siRNA, Ambion, Mulgrave, VIC, Australia) were transfected into the cells using the Amaxa Nucleofector Kit for mNSC (LONZA, Mount Waverley, VIC, Australia) according to the manufacturer's manual. The knockdown efficiency of the siRNAs was validated by qPCR. The siRNA sequences are listed in Supplementary Table S9.

### Chromatin immunoprecipitation

ChIP assays were carried out as described by Sandoval *et al.*^60^ Cells were fixed with DSP (Thermo Scientific, Waltham, MA, USA) for 45 min before fixing with 1% formaldehyde for 10 min. Glycine (0.2 mM) was used to quench the reaction. After cell lysis, chromatin extracts were sonicated (Bioruptor, Denville, NJ, USA) to fragments of ~200–500 bp and pre-cleared by Dynabeads Protein A (Invitrogen). The pre-cleared chromatin extract was incubated with C-terminal APP antibody (Invitrogen, cat no. 512700) overnight at 4 °C, and then it was incubated with Dynabeads Protein A for 4 h at 4 °C. The beads were then washed, and the chromatin–protein–antibody complexes were eluted. After treatment with Proteinase K (Finnzymes, Waltham, MA, USA), the eluted DNA was purified by the PCR purification kit (Qiagen, Chadstone, VIC, Australia). qPCR was performed to determine the fold enrichment of immunoprecipitated DNA. qPCR reaction mixes were assembled using the SYBR Green master mix (Roche, Indianapolis, IN, USA). The reactions were performed on the LightCycler 480 System (Roche) using following cycling parameters: 95 °C for 10 min, and then 45 cycles of 95 °C (10 s), 60 °C (10 s) and 72 °C (10 s) followed by a melting curve analysis. All reactions were performed with three technical replicates. Fold change values were normalized against input DNA and compared with the rabbit IgG control. The *gapdh* promoter region was selected as the negative control. The primer sequences for qPCR are listed in the Supplementary Table S9.

### Functional annotation clustering analysis

The validated target genes of the 207 miRNAs were retrieved from the miRWalk (http://www.umm.uni-heidelberg.de/apps/zmf/mirwalk/mirnatargetpub.html), and 1471 validated genes, which were targeted by 93 of miRNAs, were found. The 93 control miRNAs, which were supposed to be not influenced by AICD, were randomly selected from the 1522 miRNAs with validated target genes, and these miRNAs were not included in the two ChIP-seq data sets. The random selection was performed by the Excel (Microsoft, Redmond, WA, USA). The validated target genes of control miRNAs were also retrieved from miRWalk. The Functional annotation analysis was performed in the David Functional Annotation Clustering Analysis website (http://david.abcc.ncifcrf.gov/home.jsp), using the default setting of the medium classification stringency. The *P*-values (with Benjamini correction) of GO terms were compared.

### Lentivirus construction, production and transduction

The second-generation, self-inactivating bicistronic lentiviral transfer vector, pWPI (kindly provided by D. Trono), was used to produce lentivirus for transfection. The open reading frames for human AICD59 were cloned upstream of an IRES-eGFP cassette by blunt-end ligation to generate the vectors pWPI-AICD-IRES-eGFP. Viral stocks were generated as previously described by Siatskas *et al.*^61^ When the cells had grown to ~70% confluency, pWPI-AICD-IRES-eGFP lentiviral stock was added to the cell culture medium with 4 *μ*g/ml protamine sulfate. The cells were incubated with lentivirus overnight before changing the medium.

### Statistical analysis

Quantitative data are expressed as mean±S.E.M. Statistical significance between any two groups was determined by the two-tailed Student's *t*-test or one-way ANOVA analysis using the GraphPad Prism5 (GraphPad software, La Jolla, CA, USA). *P*-values less than 0.05 were considered significant.

## Figures and Tables

**Table 1:** Overlapped miRNA hits of the top 20 peaks from 2 ChIP-seq data sets

**Description**	**Chr**	**Start**	**End**	**Peak score**	**Annotation**	**Distance to SSS (bp)**
mir-3687^a^	chr21	9825273	9827785	3100	TTS	321
mir-663	chr20	26188340	26191020	1783.49	Promoter-TSS	−733
mir-548f-5	chr13	36531932	36532876	292.21	Intron	−17000
mir-122	chr18	56179601	56180702	215.02	Intron	61798
let-7a-1	chr9	96927827	96929643	212.295	Intergenic	−9435
mir-3910-2	chr9	94472980	94474733	210.17	Intergenic	−75228
mir-592	chr7	125691975	125692956	188.995	Intergenic	1005759
mir-3648^a^	chr21	9698762	9700581	188.45	Intergenic	−126353
mir-193a	chr17	29876126	29877625	186.215	Intergenic	−10141
mir-3943	chr7	43236886	43238815	181.425	Intron	47413
mir-1538	chr16	69599285	69600694	172.74	Promoter-TSS	−136
mir-595	chr7	157939829	157940996	170.62	Intron	385055

a mir-3687 and mir-3648 are clustered in the genome, and two different binding peaks are found for them

## Abbreviations

APP: Amyloid precursor protein
A*β*: amyloid beta peptide
AD: Alzheimer's disease
AICD: APP intracellular domain
miRNA: microRNA
NSC: neural stem cell
ChIP-seq: chromatin immunoprecipitation coupled with deep DNA sequencing
TTS: transcription terminal site
TSS: promoter-transcription start site
SSS: stem–loop start sites
FAC: functional annotation clustering
ENCODE: Encyclopedia of DNA Elements
UTR: untranslated region
pri-miRNA: primary miRNA
SCF: SKP1-CUL1-F-box protein
LFC: log2-transformed gene expression fold changes
CDF: cumulative distribution function
qPCR: quantitative PCR
