# Supplementary material for: APP intracellular domain acts as a transcriptional regulator of miR-663 suppressing neuronal differentiation
Source: Cell Death Dis. 2015 Feb 19;6(2):e1651–. doi: 10.1038/cddis.2015.10 (PMC4669786; doi:10.1038/cddis.2015.10)
Supplement: Supplementary Information [file cddis201510x1.pdf]

Supplemental Information

Materials and Methods

RNA extraction and miRNA assay

Total RNA was extracted from cells using standard Trizol (Invitrogen) method with small modifications: the RNA was precipitated by isopropanol at -20°C for more than 2 hours then washed with ice cold 80% ethanol. The concentration and integrity ($A_{260/280}$ ratio) of the RNA samples were estimated using the Nanodrop (Thermo-fisher Scientific). For miRNA expression, miR-3648, miR-3687 and miR-595 were analyzed by Taqman miRNA qPCR assays (Applied Biosystems), and other miRNAs were analyzed by miRCURY LNA™-enhanced miRNA qPCR assays (EXQION). For the Taqman miRNA qPCR assay, cDNA was synthetized using the Taqman miRNA assay kit (Applied Biosystems) according to the standard manual. The qPCR mixes were assembled using the Taqman universal PCR master Mix II (Applied Biosystems), and the cycling parameters were 50 °C (2 min), 95 °C (10 min), and then 40 cycles of 95 °C (15 s) and 60 °C (60 s). For the LNA™-enhanced miRNA qPCR assay, cDNA was synthetized using the miRCURY LNA™ Universal RT microRNA PCR, Polyadenylation and cDNA synthesis kit (EXQION) according to the standard manual. The qPCR mixes were assembled using miRCURY LNA™ Universal RT microRNA PCR, SYBR Green master mix (EXQION), and the cycling parameters were 95 °C (10min), and then 40 cycles of 95 °C (10 s) and 60 °C (60 s) followed by a melting curve analysis. All the reactions were performed on the LightCycler® 480 System (Roche), with three technical replicates. The U6 gene was selected as an internal control for miRNA expression analysis, and the comparative C_T ($\Delta\Delta C_T$) method was used for calculating relative quantitation of expression.

Flow cytometry analysis

After 4-5 days differentiation, RVM cells were detached with Accutase (Merk-Millipore) for 10 minutes in 37°C, washed once with phosphate-buffered saline (PBS), then fixed and stained with the Alexa Fluor® 647 Mouse anti-βIII-tubulin and Alexa Fluor® 488 Mouse

anti-GFAP antibodies (BD Pharmingen) according to manufacturer's manual. Cells were analyzed using a FACSCanto II flow cytometer (BD Biosciences).

Immunocytochemistry and quantification

Immunocytochemistry on the 4-5 days differentiated RVM cells were performed as we have previously described.[1] Mouse anti-βIII-tubulin antibody (Sigma-Aldrich) and rabbit anit-GFAP antibody (DAKO) were used as primary antibodies. At least 12 microscopic fields (x 100) and approximate 5000 differentiated cells for each biological sample were counted for the quantification.

Microarray analysis

Microarrays were designed and analyzed as described by Lim et al., (2005).[2] RVM cells were transfected with miR-663 mimics and control mimics, seeded in the RVM medium and total RNA was collected at 12 and 24 hours post transfection. RVM cells infected with AICD and vector control lentivirus were seeded in proliferation medium for 48 hours, and then cultured in RVM medium for another 24 hours before total RNA collection. Total RNA was collected by Trizol, purified using the RNeasy kit (Qiagen), then processed and hybridized to Agilent SurePrint G3 Human Gene Expression 8x60K v2 Microarrays. Four biological replicates were performed for statistical analysis. Data were analysed using Agilent GeneSpring GX software. For qPCR validation of gene expression, the first strand cDNA was synthesized by the SuperScript^{TM} III RT system (Life technologies) using the radom hexamers. The qPCR reactions were performed as described in the ChIP method. The GAPDH was used as the internal control for gene expression analysis. The primer sequences for qPCR are listed in the Table S9.

Luciferase reporter assay

Luciferase reporter assay was designed and analyzed as previously described.[2] The wild-type and mutant 3'UTR regions possessed the 6-7nt seed matches were cloned into the 3'end of

firefly luciferase ORF of the dual-luciferase vector pmirGLO (Promega), and the 5'UTR regions were cloned into the 5'end of firefly luciferase ORF of the dual-luciferase vector piCheck-2 (Promega). The UTR sequences and restriction enzyme sites are described below. 2.5 ug plasmid vector and 40 pmol miR-663 mimics were co-transfected into the RVM cells using the Amaxa Nucleofector Kit for mNSC (LONZA) according to manufacturer's manual. Cell lysates were collected at 24 h post transfection, and the luciferase activity was read on the CLARIOstar double injector microplate reader (BMG LABTECH) using the Dual-luciferase report assay system (Promega).

Sequences inserted into the luciferase reporter vectors

Seed matches are shown bold and underlined. All the seed sites are mutated from CCCCGCC to GGGGCGG.

1. CDK6-5UTR (Nhe I) 229bp

GCTAGCggcggcaccagggttacgccagccccgcggggaggtctctccatccagcttctgcagcggcgaaagccccagcgcc
cgagcgcctgagccggcggggagcaagtaaagctagaccgatctccggggagccccggagtaggcgagcggcggccgccagct
agttgagcgcacc**ccccgcc**cgccccagcggcgccgcggcgggcggcgtccaggcggcGCTAGC

2. CDK6-3UTR (SacI + XhoI) 512bp

GAGCTCcctttgagtggcagacactcgacggtgcctgcccaactttcctcctgagtggcaagcagatgagtctcagtaattcatac
tgaaccaaaatgccacatacactaggggcagtcagaaactggctgagaaatc**ccccgcc**tcattcgcccctctgctcccaggaactag
agtccagttaaagcccctatgcgaaaggccgaattccaccccagggtttgttataacagtggccagtctgaaccccatttgctcgtgctc
aaaacttgattcccacttgaaagccttccgggcgcgctgcctcgttg**ccccgcc**cctttggcaggagagaggcagtgggcgaggccg
ggctgggg**ccccgcc**tcccactcacctgccggtgcctgaaattatgtgcggccccgcgggctgctttccgaggtcagagtgccctgct
gctgtctcagaggcatctgttctgcaaatcttaggaagaaaaatgtccctagtagcaaacgggtgtCTCGAG

3. FBXL18-3UTR-1 (SacI + XhoI) 262bp

GAGCTCgcccccctcctgcctgtgctcccagctcaccccacacctagctgctatttattctcctgaccccccttcccggccctgcagcc
ccgtgtcccgcagcctccg**ccccgcc**tcctgctccacgtcaccaggcaacactcggctccaccaggcttccgaaggtggcccagag
caggcacttgagcctgatgacccagagcaaagctgcctttctgggccttgagtactcctttctgctatggaaggcttttcttgCTCGA
G

4. FBXL18-3UTR-2 (Xba I+ SalI) 262bp

TCTAGAaacaacaaaaagcagaaacgctgaaggggtcggtttacgggaaaaccgcctgtcagaacacttggctactcctacccc
agatcagtggacctgggaatgagggttggtcccgggaggcttttctccaagctgttgccaccaga**cccgcc**atgggaaccctggcca
cagaagcctcccggggagtgagccagagcctggaccgctgtgctgatgtgtctggggtggagggagggtggggagtgtgcaaggg
GTCGAC

5. PTP4A1-3UTR (SacI + XhoI) 562bp

GAGCTCgtggtttgcagcatgattccttccagtcgataaatcggaatctctctcgctcccacccccttcttaacttcaggcttcctgcatc
ccggagcactcccggcagccccttccctc**ccccgcc**ccggggatgctccgactcggcgcttagccattcatcaaccggttcacaccg
gcggcggccgccgcggagtgacgtccggagggggcgggcctccgc**ccccgcc**tgtcggctcctggcccgcggttccaggccgc
gattggtggctggagggttgcacgtcgcgccggctataaaggggagggcttgtgacgcaagggcgcctcggcgcgtgtattggctcc
ttcggctgcgggccggctcggctacgcgctctgctccgagccgctcactgcatggtagagtctggtgc**ccccgcc**gccgcctgcatc
gccgccaccgccgctccgccacgaccaccgccgcctcctgccctgcagccaccgccaccgcctgtgtcgccgccgcctcgggacc
ggctgtatgattaggccacaatcttcaatgagtaaacCTCGAG

6. ZFAND3-5UTR (Nhe I) 227bp
GCTAGCccgtctccgcaggccgagtggtgcggcccgcctccagctgaccggcctggaatcccggctccgagccccggactcg
cgcccgcccgcgcgcccgctccttccccctcc**ccccgcc**ccgagccccccgacgccgccgccaccgcctcctcagagcggggcc
cgggcccagccgccgccaccgctgccgccgccgagctccgccgccgccgagcaccGCTAGC

7. ZFAND3-3UTR-1 (SacI + XhoI) 312bp
GAGCTCcagagctggaaatggggggtgggggacagattcttacggaaatttttttacctgacttgctatgaaaaaactcatcacac
aagaagagaaacagtaacctcactttgaaaattagctccactcaagactagtccacgaacgagacccgccttttctacacaggatccaa
ggtcacgagaagcagccagagtg**ccccgcc**tccgccggctctggtctgccattcgccagtgcagggatctggcacggaccagatgt
ggcgaatggcagcacagcgcggtggctgggtctgcacactggcctCTCGAG

8. ZFAND3-3UTR-2 (Xba I+ SalI) 262bp
TCTAGAccactgctgtcaagccacagcccttggccaccatacgggccatcctcagtgaggcagccccccataggcttccgccaa
gctctggtcccgaagaggctgtgcgagcccttcccggccctccccagggcc**ccccgcc**ccctcctctgcctgctgcgtggaggcagc
catgggaaggagcccaggggagctggcctgggggagcgaagcccatgttcgcttcctgacttagagctgggggggggtggggggtg
GTCGAC

BrdU incorporation assay

Transfected RVM cells were seeded on coverslip at a density of 30000 cells /cm^2. At 24 and 48 hours after transfection, cells were incubated with 10 μM BrdU (Sigma-aldrich) for 2 hours. Cells were fixed with cold 70% ethanol, treated with 1.5N HCl, followed by immunostaining with anti-BrdU antibody according to manufacturer's manual (Cell Signaling Technology).

Figure S1

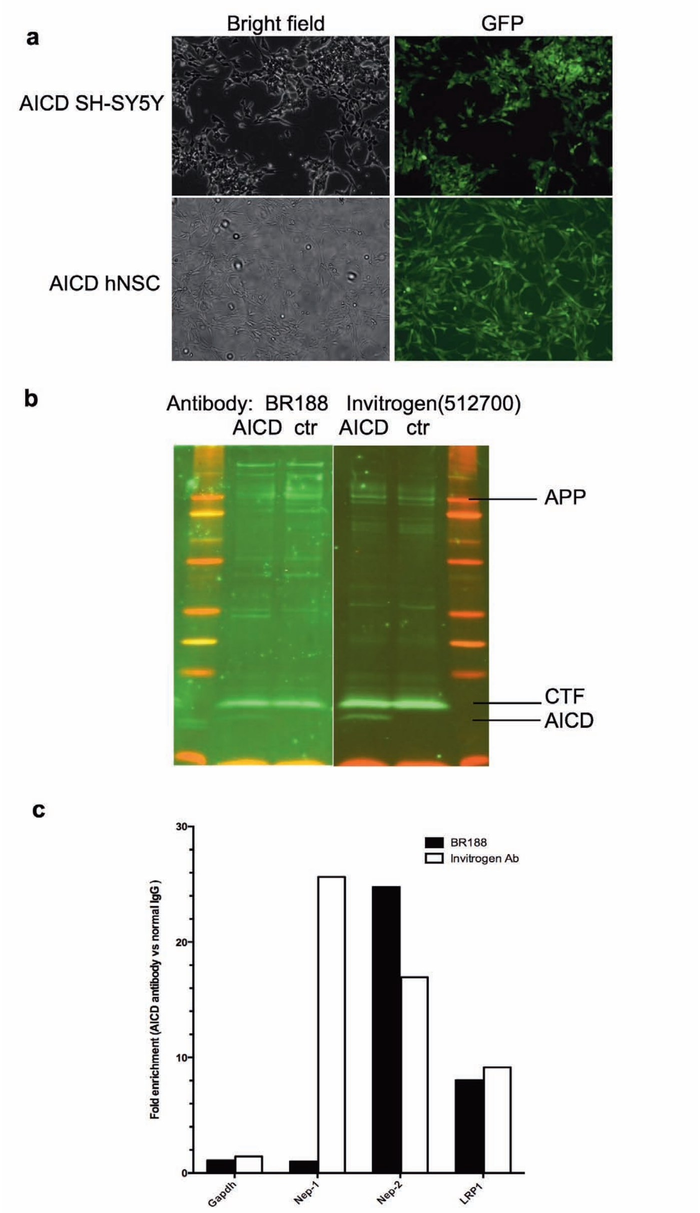

Figure S1. Validation of the AICD over-expressed cells and AICD antibody. (**a**) The transduction efficiency after AICD-IRES-GFP lentivirus infection was evaluated roughly by the ratio of GFP positive cells. The GFP positive cells were approximately 95%. (**b**) In AICD transfected SH-SY5Y cells, the AICD peptide was increased significantly comparing to the control. In addition, western blot analysis showed that the APP C-terminal antibody from Invitrogen had higher specificity and reactivity than the BR188 APP C-terminal antiserum. (**c**) ChIP pre-experiment checking the enrichment of positive control gene promoters, such as Neprilysin (NEP-1 and NEP-2) and LRP1[3, 4] confirmed that the Invitrogen AICD antibody could be used in ChIP assay.

Figure S2

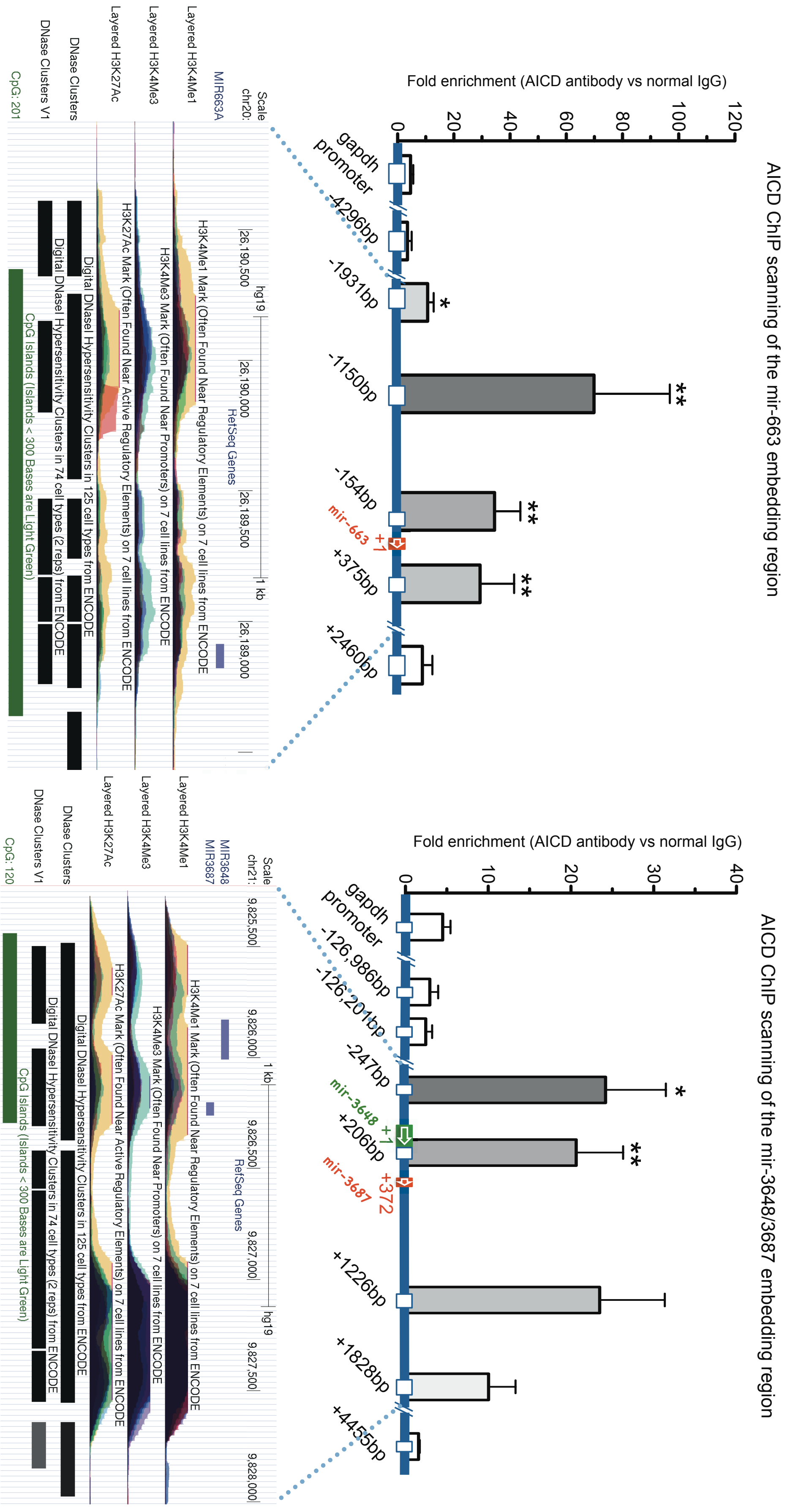
AICD ChIP scanning of the mir-663 embedding region
Fold enrichment (AICD antibody vs normal IgG)
0
20
40
60
80
100
120
gapdh promoter
-4296bp
-1931bp
-1150bp
-154bp
mir-663 +1
+375bp
+2460bp
*
**
**
**
Scale
chr20:
hg19
1 kb
26,190,500
26,190,000
26,189,500
26,189,000
MIR663A
RefSeq Genes
Layered H3K4Me1
H3K4Me1 Mark (Often Found Near Regulatory Elements) on 7 cell lines from ENCODE
Layered H3K4Me3
H3K4Me3 Mark (Often Found Near Promoters) on 7 cell lines from ENCODE
Layered H3K27Ac
H3K27Ac Mark (Often Found Near Active Regulatory Elements) on 7 cell lines from ENCODE
DNase Clusters
Digital DNaseI Hypersensitivity Clusters in 125 cell types from ENCODE
DNase Clusters V1
Digital DNaseI Hypersensitivity Clusters in 74 cell types (2 reps) from ENCODE
CpG: 201
CpG Islands (Islands < 300 Bases are Light Green)
AICD ChIP scanning of the mir-3648/3687 embedding region
Fold enrichment (AICD antibody vs normal IgG)
0
10
20
30
40
gapdh promoter
-126,986bp
-126,201bp
-247bp
mir-3648 +1
+206bp
mir-3687 +372
+1226bp
+1828bp
+4455bp
*
**
Scale
chr21:
1 kb
hg19
9,825,500
9,826,000
9,826,500
9,827,000
9,827,500
9,828,000
MIR3648
MIR3687
RefSeq Genes
Layered H3K4Me1
H3K4Me1 Mark (Often Found Near Regulatory Elements) on 7 cell lines from ENCODE
Layered H3K4Me3
H3K4Me3 Mark (Often Found Near Promoters) on 7 cell lines from ENCODE
Layered H3K27Ac
H3K27Ac Mark (Often Found Near Active Regulatory Elements) on 7 cell lines from ENCODE
DNase Clusters
Digital DNaseI Hypersensitivity Clusters in 125 cell types from ENCODE
DNase Clusters V1
Digital DNaseI Hypersensitivity Clusters in 74 cell types (2 reps) from ENCODE
CpG: 120
CpG Islands (Islands < 300 Bases are Light Green)

Figure S2. Encyclopedia of DNA Elements (ENCODE) and CpG islands are enriched in the mir-663 and mir-3648/mir3687 embedding region. The ENCODE elements include H3K4Me1 mark, H3K4Me3 mark, H3K27Ac mark, Digital DNaseI Hypersensitivity Clusters. The ChIP results performed in the AICD over-expressing RVM cells are also shown. As we can see, the positions of significant enrichment of AICD are just located within the peak regions of the H3K4Me1 mark, H3K4Me3 mark and H3K27Ac mark**.** In addition, these regions are overspread with DNase Clusters and/or CpG ilands.

Figure S3

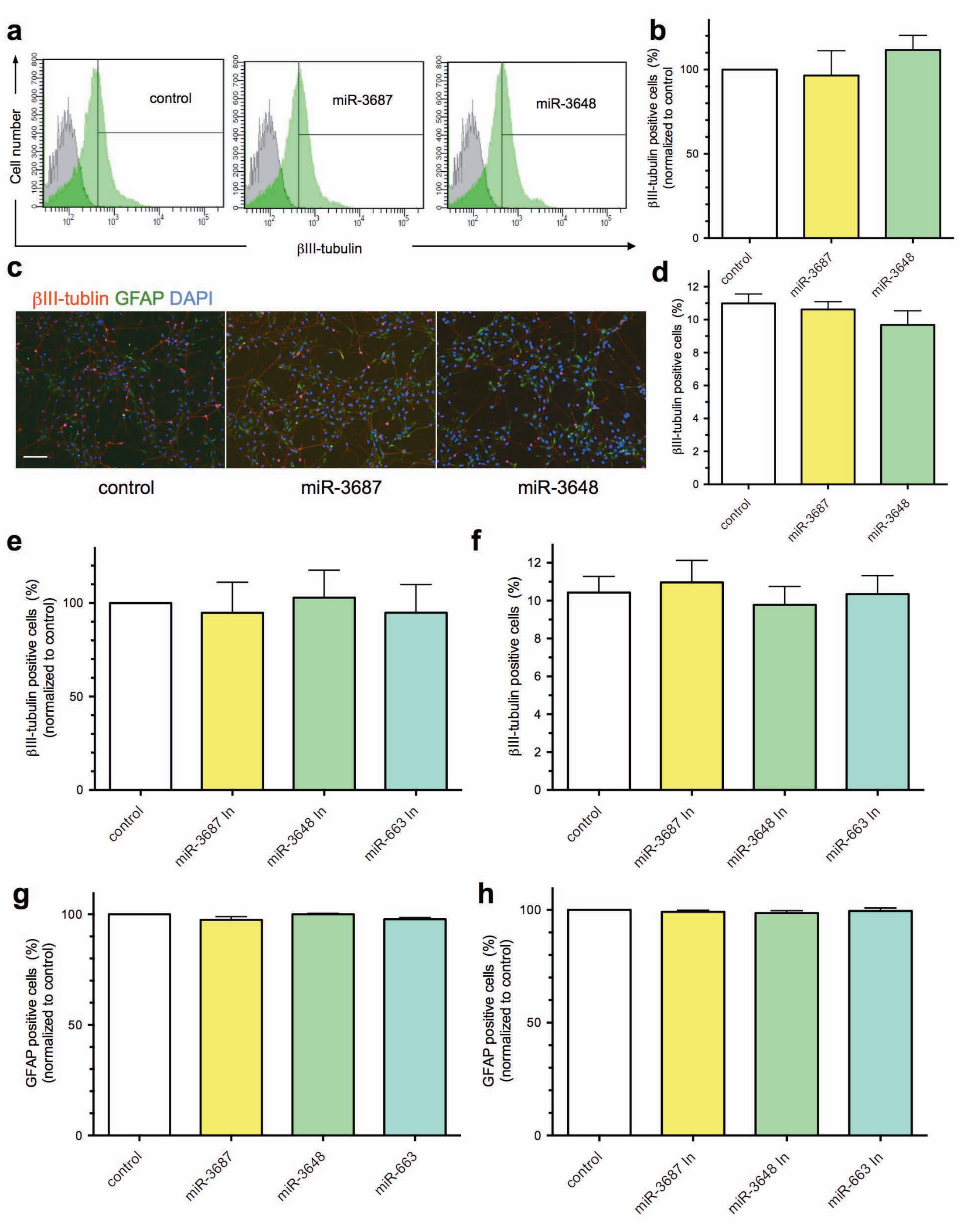
a
Cell number
control
miR-3687
miR-3648
βIII-tubulin
b
βIII-tubulin positive cells (%) (normalized to control)
control
miR-3687
miR-3648
c
βIII-tublin GFAP DAPI
control
miR-3687
miR-3648
d
βIII-tubulin positive cells (%)
e
βIII-tubulin positive cells (%) (normalized to control)
miR-3687 In
miR-3648 In
miR-663 In
f
g
GFAP positive cells (%) (normalized to control)
miR-663
h

Figure S3. miRNAs and NSC differentiation. (**a** to **d**) mir3687 and mir3648 mimics did not modulate the hNSC neuronal differentiation. (**a**) Representative histograms and (**b**) quantification analysis result of βIII-tubulin detection by flow cytometry in hNSCs transfected with mir3687, mir3638 and control mimics after neural cell differentiation, respectively. (**c**) Representative images and (**d**) quantification analysis result of βIII-tubulin detection by immunohistochemistry in hNSCs transfected with mir3687, mir3638 and control mimics after neural cell differentiation, respectively. Scale bar = 200 um. (**e** and **f**) Inhibitors of mir663, mir3687 and mir3648 could not influence the neuronal differentiation significantly. Quantification analysis result of βIII-tubulin detection by (**e**) flow cytometry and (**f**) immunohistochemistry in hNSCs transfected with miRNA inhibitors after neural cell differentiation. (**g** and **h**) Neither the mimics nor the inhibitors of these four miRNAs transfection changed the percentage of astrocytes after neural differentiation. Quantification analysis result of GFAP positive cells by flow cytometry in hNSCs transfected with (**g**) miRNA mimics and (**h**) inhibitors after neural cell differentiation. Quantification data were analyzed from at least three independent experiments (mean ± s.e.m).

Figure S4

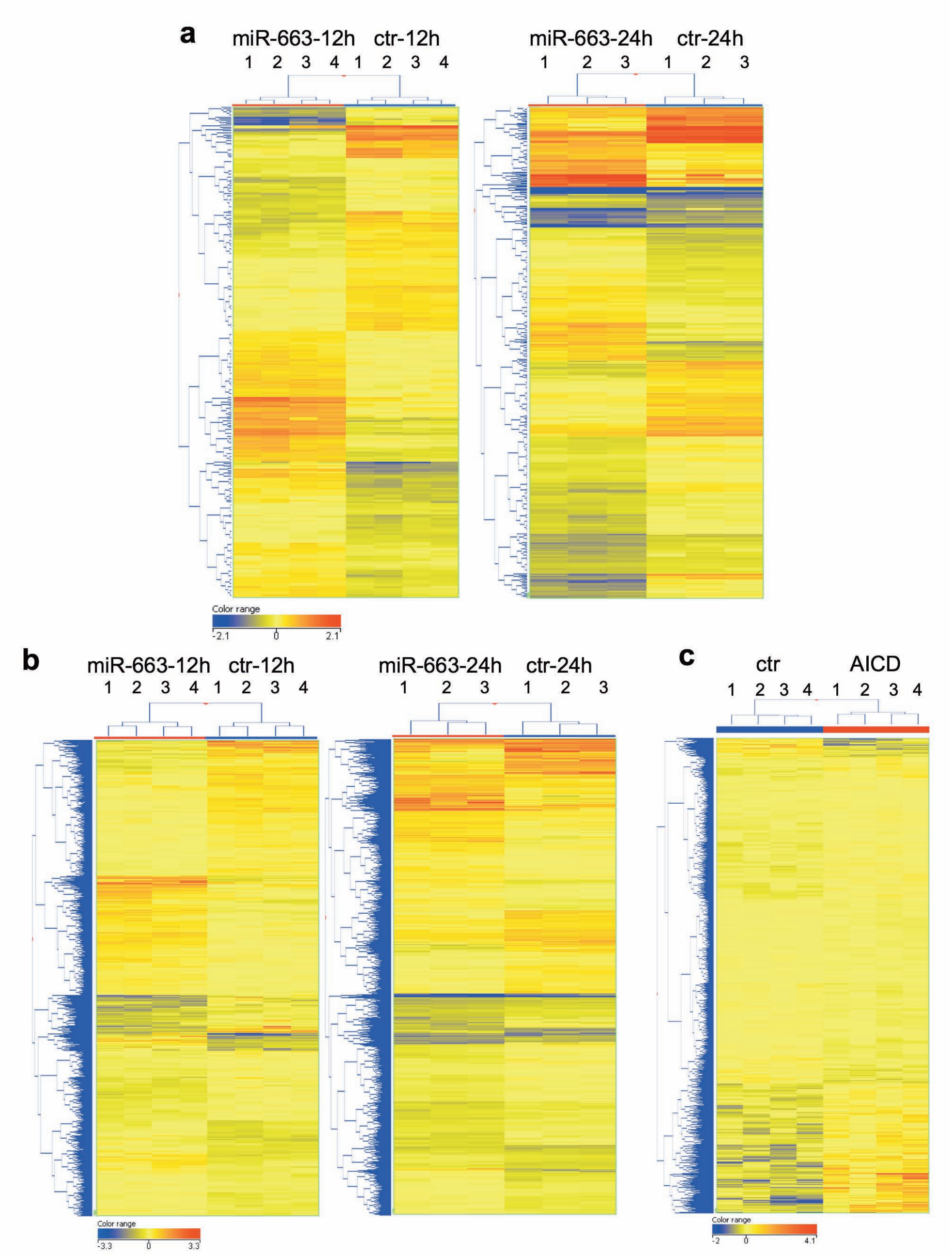

Figure S4. Heatmaps of microarrays analysis in this study. (**a**) Hierarchical clustering of microarray gene expression profiles at 12 and 24h after mir663 transfection ($P<0.001$). hNSCs were transfected with mir663 mimics or the control ($n \geq 3$). (**b**) Hierarchical clustering of microarray gene expression profiles at 12 and 24h after mir663 transfection ($P<0.05$). hNSCs were transfected with mir663 mimics or the control ($n \geq 3$). (**c**) Hierarchical clustering of microarray gene expression profiles after AICD transfection ($P < 0.05$). hNSCs were transduced with AICD or the control ($n = 4$).

Figure S5

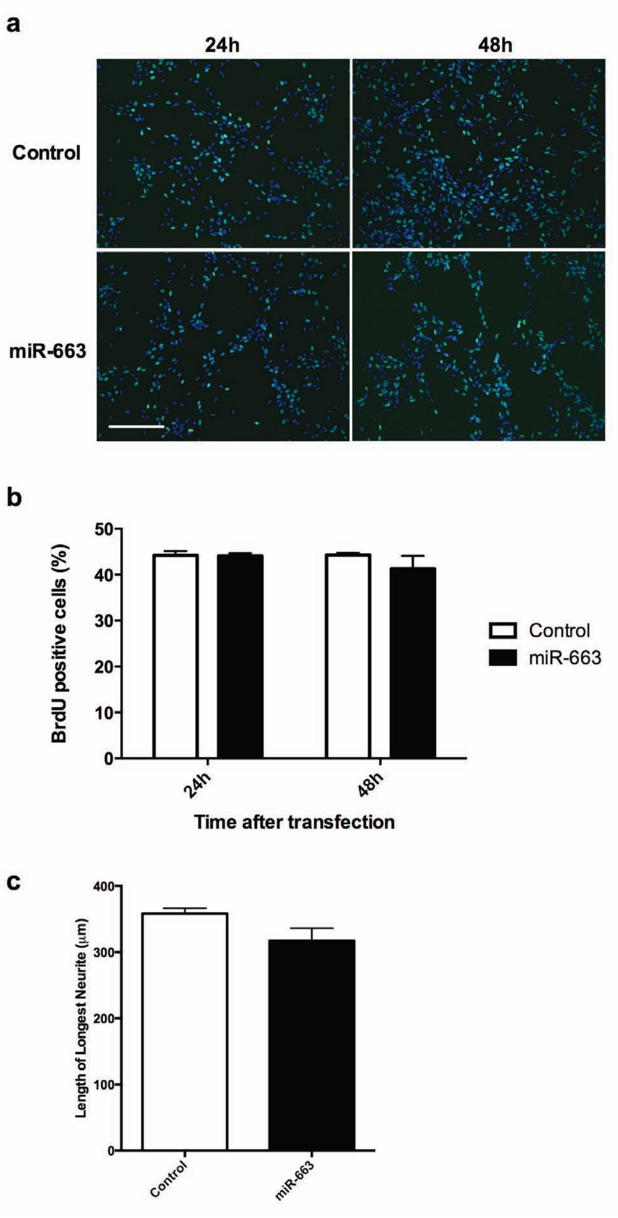

Figure S5. Proliferation and neurite length assay after miR-663 overexpression. (**a**) Representative images of BrdU (Green) immunostaining after miR-663 transfection in RVM cells. (DAPI = Blue; Scale bar = 200 um.) (**b**) Quantitative analysis of BrdU positive cells after miR-663 transfection in RVM cells. (**c**) The length of longest neurites was analyzed using the NeuronJ[5] in the differentiated RVM cells after miR-663 transfection. Quantification data were analyzed from at least three independent experiments (mean ± s.e.m).

Supplemental References

1. Ma QH, Futagawa T, Yang WL, Jiang XD, Zeng L, Takeda Y*, et al.* A TAG1-APP signalling pathway through Fe65 negatively modulates neurogenesis. *Nature cell biology* 2008, **10**(3)**:** 283-294.

2. Lim LP, Lau NC, Garrett-Engele P, Grimson A, Schelter JM, Castle J*, et al.* Microarray analysis shows that some microRNAs downregulate large numbers of target mRNAs. *Nature* 2005, **433**(7027)**:** 769-773.

3. Liu Q, Zerbinatti CV, Zhang J, Hoe HS, Wang B, Cole SL*, et al.* Amyloid precursor protein regulates brain apolipoprotein E and cholesterol metabolism through lipoprotein receptor LRP1. *Neuron* 2007, **56**(1)**:** 66-78.

4. Belyaev ND, Nalivaeva NN, Makova NZ, Turner AJ. Neprilysin gene expression requires binding of the amyloid precursor protein intracellular domain to its promoter: implications for Alzheimer disease. *EMBO reports* 2009, **10**(1)**:** 94-100.

5. Meijering E, Jacob M, Sarria JC, Steiner P, Hirling H, Unser M. Design and validation of a tool for neurite tracing and analysis in fluorescence microscopy images. *Cytometry Part A : the journal of the International Society for Analytical Cytology* 2004, **58**(2)**:** 167-176.
